# Glomerular podocyte dysfunction in inherited renal tubular disease

**DOI:** 10.1007/s12519-021-00417-0

**Published:** 2021-02-24

**Authors:** Li-Min Huang, Jian-Hua Mao

**Affiliations:** Department of Nephrology, National Clinical Research Center for Child Health, The Children’s Hospital, Zhejiang University School of Medicine, #57 Zhugan Lane, Hangzhou 310006, China

**Keywords:** Cystinosis, Dent disease, Gitelman syndrome, Podocyte, Renal tubule disease

## Abstract

**Background:**

Hereditary renal tubular disease can cause hypercalciuria, acid-base imbalance, hypokalemia, hypomagnesemia, rickets, kidney stones, etc. If these diseases are not diagnosed or treated in time, they can cause kidney damage and electrolyte disturbances, which can be detrimental to the maturation and development of the child. Glomerular involvement in renal tubular disease patients has only been considered recently.

**Methods:**

We screened 71 papers (including experimental research, clinical research, etc.) about Dent’s disease, Gitelman syndrome, and cystinosis from PubMed, and made reference.

**Results:**

Glomerular disease was initially underestimated among the clinical signs of renal tubular disease or was treated merely as a consequence of the tubular damage. Renal tubular diseases affect glomerular podocytes through certain mechanisms resulting in functional damage, morphological changes, and glomerular lesions.

**Conclusions:**

This article focuses on the progress of changes in glomerular podocyte function in Dent disease, Gitelman syndrome, and cystinosis for the purposes of facilitating clinically accurate diagnosis and scientific treatment and improving prognosis.

## Introduction

The renal tubule is an important part of the kidney that determines the body’s water-electrolyte and acid-base balance, reabsorbs nutrients, and concentrates or dilutes the urine. Renal tubular disease can cause hypercalciuria, acid-base imbalance, hypokalemia, hypomagnesemia, rickets, kidney stones, etc. If these diseases are not diagnosed and treated in time, they can hinder the maturation and development of the child and cause long-term recurrent kidney stones or electrolyte imbalances, exacerbating kidney impairment. Kidney tubular diseases in children are typically genetic, including Gitelman syndrome, Dent disease, and cystinosis (CTNS).

Podocytes are important intrinsic cells of the glomerulus that receive various pathological stimuli. Architecturally, the glomerulus or renal corpuscle consists of aglomerular tuft and Bowman’s capsule. The basic unit of the glomerular tuft is a single capillary. The glomerular basement membrane (GBM) provides the primary structural scaffold for the glomerular tuft. Endothelial and smooth muscle-like mesangial cells providing capillary support are located inside the GBM, whereas podocytes are attached to the outer part of the GBM [1]. Podocytes constitute the molecular and charge barrier of the glomerular filtration membrane, withstanding the force per unit area in the glomeruli. Podocyte foot processes elaborate into a highly branched interdigitating network with foot processes of neighboring podocytes. The slit diaphragm bridges the filtration slits between opposing podocyte foot processes [2], thereby establishing the final barrier to urinary protein loss [3]. The podocyte adjusts and maintains homeostasis, although excessive stress can lead to maladjustment, accompanied by complex biological changes, including loss of integrity and abnormal metabolism (the results are foot processes effacement reflected by the simplification of the foot processes structure and loss of the normal interdigitating pattern and proteinuria [4]). The latest developments in the interaction between the renal tubule and podocytes and the functional changes of podocytes in renal tubular diseases are summarized below.

## Podocyte changes in Dent disease

Dent disease is a rare X-linked recessive renal disorder that is found almost exclusively in males, manifested as renal tubular disease, hypercalciuria, and renal tubular proteinuria. Dent disease is characterized by low-molecular-weight (LMW) proteinuria, hypercalciuria, kidney stones, variable manifestations of proximal tubular dysfunction, and progressive renal failure, which ultimately leads to chronic kidney disease in adulthood [5, 6]. Dent disease may vary in clinical presentation with proteinuria alone or in combination with nephrocalcinosis or nephrolithiasis, with or without chronic kidney disease [7]. Dent disease can start in early childhood, usually before the age of ten [8, 9]. Asymptomatic cases are occasionally diagnosed in adult age, while 30–80% of patients aged between 30 and 50 years old will progress to end-stage renal disease [10–12]. In approximately 65% of patients, mutations of chloride voltage-gated channel 5 gene (*CLCN5*) are responsible for Dent disease type 1 [13, 14], while in 10–15% of patients, mutations in the oculocerebrorenal syndrome of Lowe gene (*OCRL*) cause Dent disease type 2 [15]. The remaining 25% of patients have a Dent disease phenotype, but specific genetic mutations have not been described [9, 16].

In recent years, glomerular involvement in Dent disease has been considered. Since the discovery of *CLCN5* and *OCRL* expression in the glomerular compartment, a new theory has emerged according to which these two proteins’ loss of function lead to primary glomerular cell damage [17, 18]. Glomerular damage accounted for the nephrotic-range proteinuria observed in more than 30% of patients with Dent disease [19]. *CLCN5* encodes for electrogenic chloride channel Cl^−^/H^+^ antiporter ClC-5 that is primarily expressed in proximal tubular cells, but it is also expressed in epithelial cells of ascending limb of loop of Henle and alpha intercalated cells of collecting duct [20]. Proximal tubular cells also reportedly express *CLCN5* in the brush border plasma membrane, where it is needed for LMW protein reabsorption [20]. There is increasing evidence of glomerular protein handling by podocytes [21–23]. *CLCN5* encoded protein ClC-5, plays a role in the uptake of LMW proteins through receptor-mediated proximal tubule endocytosis. Human podocytes were also demonstrated to be capable of internalizing albumin predominantly through a cubilin-amnionless mediated mechanism. Furthermore, the excess albumin environment induced an increase in *CLCN5* expression in these cells [21]. Overexpression of *CLCN5* found in biopsies of patients with proteinuria suggests that this condition may play a role in its expression, and podocytes may play a key role in albumin processing therein [17]. Similar to proximal tubular cells, the endocytosis mechanism plays a role in podocytes and in maintaining the glomerular filtration barrier [24]. The importance of endocytosis in podocyte homeostasis is confirmed [21, 25, 26].

Gianesello et al. demonstrated that human podocytes were able to internalize albumin under normal condition, suggesting that these cells are committed to protein uptake [21]. At the proximal renal tubular level, ClC-5 (encoded by *CLCN5*) and Megalin (encoded by *LRP2*) are part of a molecular complex involved in the endocytosis and re-uptake of LMW proteins and albumin. Piwon et al. showed that disruption of the mouse *CLCN5* gene causes proteinuria by strongly reducing apical proximal tubular endocytosis. Both receptor-mediated and fluid phase endocytosis are affected [27]. In addition to renal tubular dysfunction, *CLCN5* mutations may also cause podocyte dysfunction, leading to histological manifestations of focal segmental glomerulosclerosis [28–30]. Compared with the control group, *CLCN5* knock down human podocytes have a reduced proliferation rate, increased cell migration rate as assessed by the scratch test, and a defect in the endocytosis of transferrin [28]. It is reported that the increased cell migration rate is abnormal and is a sign of podocyte damage [31–34]. Glomerulosclerosis was a common finding in kidney biopsies from patients with Dent disease type 1 [35]. Bignon et al. [6] provided some evidence that focal segmental glomerulosclerosis or focal global glomerulosclerosis observed in Dent disease might be the result of primary podocyte injury independent of tubular injury.

Mutations in the *OCRL* gene cause both Dent disease type 2 and Lowe syndrome [36], suggesting a genotype-phenotype correlation [37]. The condition of patients with Dent disease type 2 is mild, and renal impairment is milder than in patients with Lowe syndrome [12]. Renal calculi in patients with Dent disease type 2 are considerably less than in patients with Dent disease type 1 [38]. The *OCRL* gene is expressed in all human cells except cells of hematopoietic origin, it is widely expressed in the kidney, including the glomerulus and most tubular segments [39, 40]. The *OCRL* gene was very recently reported to be more widely expressed in human glomeruli than *CLCN5*—the former in podocytes, mesangial cells and endothelial cells, the latter in podocytes and parietal epithelial cell (PECs) [18]. Whereas the *OCRL* is expressed in podocytes, mesangial cells and endothelial cells, *CLCN5* is expressed in podocytes and PECs [18]. *OCRL* is primarily expressed in the trans-Golgi network, early endosomes, and lysosomes (in HeLa, normal rat kidney NRK, and COS-7 cells, fibroblasts, zebrafish embryos) [41–43]. It was suggested that OCRL is involved in regulating endocytic trafficking, actin cytoskeleton dynamics, and slit diaphragm maintenance. Mutations of the *OCRL* gene could disrupt these mechanisms, thereby inducing glomerular damage [18]. Given that mutations in *CLCN5* and *OCRL* produce very similar kidney defects in human patients [44], one might expect that CLC-5 and OCRL cooperate in a similar or shared cellular process. *OCRL* is located at various positions in the sequence encoding the endocytosis pathway and is thought to play a role by coupling the endocytotic membrane with dephosphorylation of inositol 5-phosphatase [41, 45]. Preston et al. [18] showed that *OCRL* is expressed in podocytes in vivo and is able to interact with *CD2AP*, an important protein whose function is to maintain the slit dia-phragm between adjacent podocyte foot processes. Their results raise the possibility that defective *OCRL* can directly cause a glomerulopathy. The functions of *OCRL* and proton-chloride ion-exchange transporter 5 are concentrated on a shared mechanism, and their damage has a significant effect on proximal tubule endocytosis [46]. Podocyte foot process effacement was discovered in patients with Dent disease, which suggested that glomerulosclerosis in these patients might be the result of a combination of primary podocyte injury and a reaction secondary to tubulointerstitial lesions (tubulointerstitial injury was commonly present and associated with the proportion of globally sclerotic glomeruli) [35].

## Podocyte changes in Gitelman syndrome

Gitelman syndrome, also known as familial hypokalemia-hypomagnesemia, is an autosomal recessive salt loss renal tubular disease characterized by hypomagnesemia, hypocalciuria, and hyperaldosteronism, the cause of hypokalemia and metabolic alkalosis [47]. Gitelman syndrome is typically caused by mutations in the *SLC12A3* gene encoding the thiazide-sensitive NaCl cotransporter or the *CLCNKB* gene encoding the chloride channel ClC-Kb [48]. Most cases are caused by mutations in the *SLC12A3* gene, and more than 140 different *SLC12A3* mutations have been identified in patients with Gitelman syndrome. In most cases, symptoms do not appear before the age of six, and the disease is usually diagnosed during adolescence or adulthood.

According to reports, renal biopsy in patients with Gitelman syndrome showed an enlarged proximal tubule and thickened mesangium under a light microscope [49]. Similar observations were made in renal biopsies from *SLC12A3* knockout mice, showing thickened GBM in a wide range of segments, with the thickness of these irregular GBMs accompanied by podocyte foot processes. The disappearance and occasional formation of pseudocysts in podocytes confirm the potential association of glomerular defects with Gitelman syndrome [49]. The lesions observed in this case and the mouse model suggest that there may be a link between the loss of NaCl cotransporter function and podocyte dysfunction. One hypothesis is that the chronic activation of the renin-angiotensin-aldosterone pathway leads to increased systemic and local levels of angiotensin II (Ang II) and renin, which may in turn cause podocyte damage.

Mechanical stress of podocytes stimulates local Ang II synthesis by non-angiotension converting enzyme pathways that presumably involve chymase [50]. Ang II induces transforming growth factor-β1 (TGF-β1) in the various renal cells [51, 52]. TGF-β is well-known among growth factors for its potent and widespread actions. Almost every cell in the body has been shown to make some form of TGF-β, and almost every cell expresses receptors for TGF-β. TGF-β plays an important role in podocyte isolation [53–55]. One paper showed that TGF-β1 reduced nephrin expression in conditionally immortalized human podocytes [56]. Ang II has a direct effect on the integrity of the ultrafiltration barrier and reduces the cell surface and extracellular matrix of podocytes. Ang II reduces the synthesis of negatively charged proteoglycans [57, 58]. Complete nephrin (nephropathy protein)-nephrin signaling is important for podocyte survival; thus, Ang II-mediated nephrin inhibition leads to podocyte apoptosis [58]. Ang II stimulates albumin endocytosis in proximal tubule cells via Ang II type 2 receptor–mediated protein kinase B activation. However, an increase in tubular albumin reabsorption activates the tubular renin–angiotensin–aldosterone system, leading to a vicious circle [59]. In another report of Gitelman syndrome, renal biopsy showed severe non-apoptotic podocyte detachment in the glomeruli and thickening of the intimal fibers of the small arteries [60].

## Podocyte changes in cystinosis

CTNS is an autosomally recessive lysosomal storage disease caused by a deficiency of cystinosin (a lysosomal membrane cystine transporter). This defect causes cystine to crystallize in the lysosomes of many tissues, particularly in the kidneys and cornea. Renal manifestations of CTNS include Fanconi syndrome, mild proteinuria, and progressive renal failure. CTNS is caused by a pathogenic mutation in the human *CTNS* gene encoding cystinosin [61]. The kidney is initially affected by widespread proximal tubular dysfunction, which rapidly affects the glomeruli and progresses into end-stage renal failure and multiple organ dysfunction. The accumulation of cystine may involve abnormal nuclear division, with a lack of cytokinesis in the injured podocytes resulting in the appearance of multinucleation [62]. This provides further evidence for the diagnosis of cystine disease. Sharma et al. found that a patient had extensive giant cell transformation of glomerular podocytes, accompanied by focal atrophy and expansion of renal tubules [63].

Studies have shown that the *CTNS* gene is essential for the function of zebrafish anterior renal podocytes and proximal renal tubules. Anterior kidneys of *CTNS*-knockout zebrafish show enlarged lysosomes in proximal renal tubular cells, part of podocytes disappear, and slit membrane stenosis [64]. Podocytes can move in the glomerulus and break through the Bowman's capsule and be, rapidly replaced by a stellate cell. This change in podocyte movement is considered to be the basis of the disappearance of the foot process and proteinuria [65]. The number of podocytes in the urine of patients with cystine disease is considerably greater than in the urine of normal subjects. The impaired ability of cells to adhere to substrates may be the cause of mass loss of glomerular podocytes, resulting in damage in this region. Increased movement of podocytes lacking cystinosin is associated with increased phosphorylation of protein kinases [66]. Protein kinase 1 is predominantly expressed in the proximal tubules of the kidney, while protein kinase 2 is mainly expressed in the glomeruli, which protects the glomeruli and prevents podocyte dedifferentiation and death [67].

## Podocyte injury mechanism in renal tubular diseases

### Mechanical injury

Podocyte detachment in Gitelman syndrome may be related to obstruction of the nephron and decreased protein expression. Mechanical stretching and TGF-β stress may induce podocyte apoptosis or separation from the GBM [68]. In cystine disease, cystine crystals are deposited in podocyte lysosomes, resulting in the appearance of multinucleation, changes in the cytoskeleton, and enhanced podocyte motility, etc.

### Gene defects cause podocyte damage

*SLC12A3* gene mutation can cause podocyte non-apoptotic detachment. *CLCN5*-mutated podocytes undergo endocytosis and reduced proliferative ability, accompanied by enhanced migration ability [28]. Furthermore, these mutations cause changes in the podocyte cytoskeleton, damage podocyte adhesion sites, and enhance the mobility of individual podocytes, causing detachment and death.

### Pro-inflammatory factors and cytokines

Ang II can induce proteinuria through hemodynamic and non-hemodynamic mechanisms involving vascular endothelial growth factor and TGF-β1 [69]. Contrary to physiological conditions, the pathophysiology of podocyte injury is generally related to increased expression of TGF-β, which plays an important role in podocyte isolation [70]. In response to TGF-β and other TGF-dependent stimuli, mature podocytes undergo dedifferentiation, resulting in the disappearance of foot processes.

### Epigenetics

**Fig. 1:**
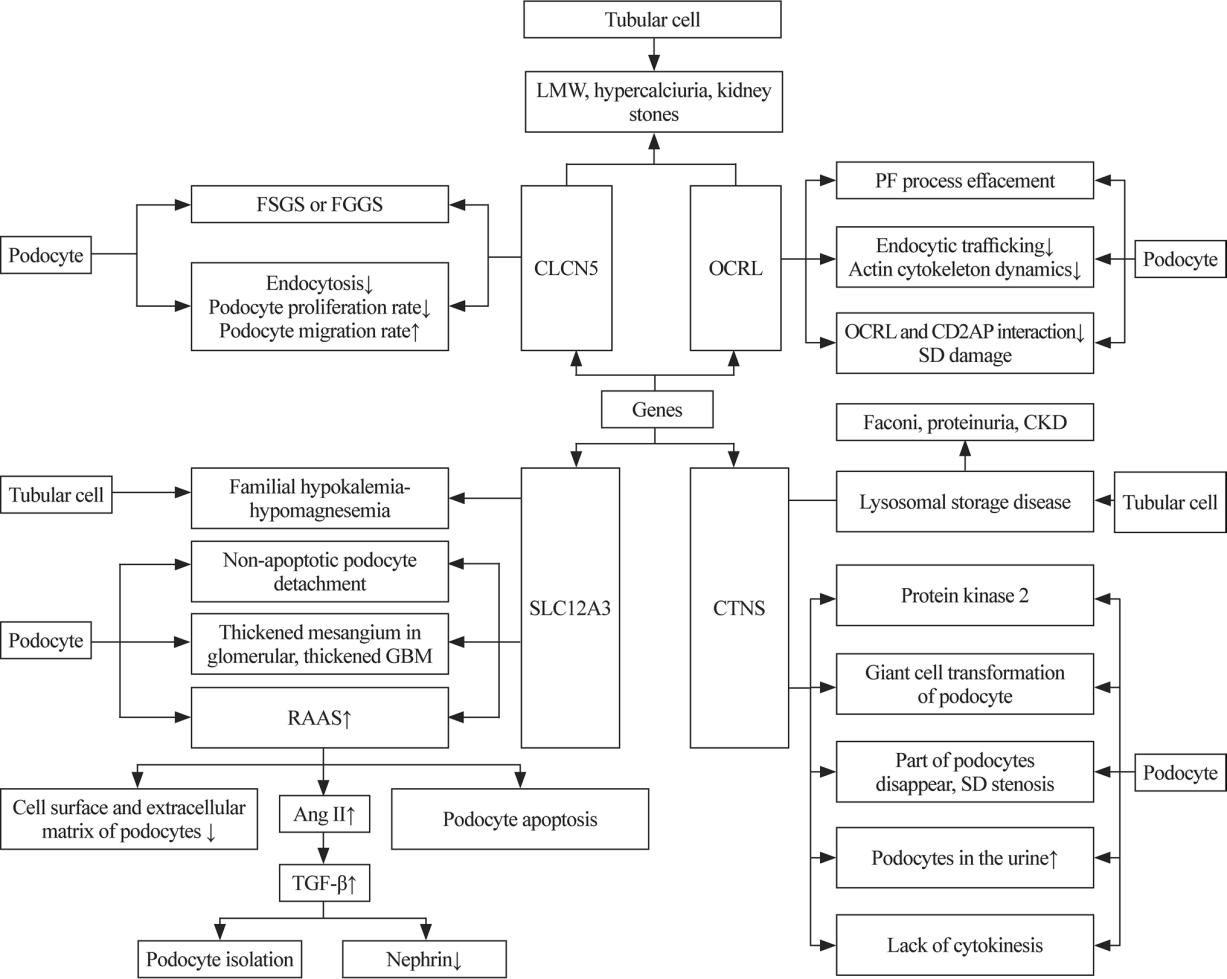
Podocyte injury mechanism in renal tubular diseases. *FSGS* focal segmental glomerulosclerosis, *FGGS* focal global glomerulosclerosis, *LMW* low-molecular-weight, *CLCN5* chloride voltage-gated channel 5 gene, *CD2AP* CD2-associated protein, *GBM* glomerular basement membrane, *RAAS* renin–angiotensin–aldosterone system, *CTNS* cystinosis, *CKD* chronic kidney disease, *TGF-ß* transforming growth factor-β, *PF* podocyte foot, *SD* slit diaphragm, *Ang II* angiotensin II

A decrease in sirtuin 1 (Sirt1) expression in the renal tubules causes a decrease in Sirt1 levels in the glomeruli, suggesting that molecular changes in the renal tubules induce phenotypic changes in the glomeruli and podocytes, with the disappearance of more podocytes. Additionally, this reveals the role of nicotinamide mononucleotide as a mediator of interaction between renal tubular cells and podocytes, as nicotinamide mononucleotide derived from renal tubular cells is absorbed by podocytes [71] (Fig. 1).

## Conclusions

To study podocyte damage and its mechanism in inherited renal tubular disease, from a clinical perspective, it is useful to explain the phenotype of glomerular lesions in patients with renal tubular disease, to guide clinical treatment and prognosis. Research into the glomerulus-tubule dialogue and mutual feedback pathways should be further investigated. Moreover, further research on the specific roles and molecular mechanisms involved in functioning of glomerulus, renal tubules, etc., should be expanded.

This article reviewed the glomerular podocyte damage caused by the three inherited renal tubular diseases, Gitelman syndrome, Dent disease, and cystine disease. However, other renal tubular diseases may affect glomerular podocyte morphology, abnormal function, and quantity, and affect the phenotype and prognosis of patients. From this perspective, actively exploring podocyte lesions of the renal tubular disease may have important clinical significance.
